# Gray matter correlates of cognitive ability tests used for vocational guidance

**DOI:** 10.1186/1756-0500-3-206

**Published:** 2010-07-22

**Authors:** Richard J Haier, David H Schroeder, Cheuk Tang, Kevin Head, Roberto Colom

**Affiliations:** 1 University of California, School of Medicine (Emeritus), Irvine CA, USA; 2 Johnson O'Connor Research Foundation, Chicago IL, USA; 3 Mt. Sinai Medical Center, School of Medicine, New York NY, USA; 4 University of California, School of Medicine, Irvine CA, USA; 5 Universidad Autónoma de Madrid, Spain

## Abstract

**Background:**

Individual differences in cognitive abilities provide information that is valuable for vocational guidance, but there is an ongoing debate about the role of ability factors, including general intelligence (*g*), compared to individual tests. Neuroimaging can help identify brain parameters that may account for individual differences in both factors and tests. Here we investigate how eight tests used in vocational guidance correlate to regional gray matter. We compare brain networks identified by using scores for ability factors (general and specific) to those identified by using individual tests to determine whether these relatively broad and narrow approaches yield similar results.

**Findings:**

Using MRI and voxel-based morphometry (VBM), we correlated gray matter with independent ability factors (general intelligence, speed of reasoning, numerical, spatial, memory) and individual test scores from a battery of cognitive tests completed by 40 individuals seeking vocational guidance. Patterns of gray matter correlations differed between group ability factors and individual tests. Moreover, tests within the same factor showed qualitatively different brain correlates to some degree.

**Conclusions:**

The psychometric factor structure of cognitive tests can help identify brain networks related to cognitive abilities beyond a general intelligence factor (*g*). Correlates of individual ability tests with gray matter, however, appear to have some differences from the correlates for group factors.

## Findings

Individual differences in cognitive abilities provide information that is valuable for vocational guidance, but there is an ongoing debate about the role of a general factor of intelligence, "*g*", that accounts for common variance among cognitive tests [1]. On the one hand, in large samples, *g *predicts job performance very well [2-4]; on the other hand, specific cognitive ability tests provide useful information for individuals, especially for job choice [5-7]. On a practical level, psychometric testing used for vocational guidance assesses both general intelligence and specific cognitive abilities [8].

A new direction in research into the nature of intelligence and cognitive abilities is the use of neuroimaging to identify brain parameters that may help account for individual differences in psychometric test scores [9-11]. Several recent neuroimaging studies of brain structure, for example, relate variation in regional gray and white matter to performance on tests with high *g*-loadings [12-14]. Colom et al. [15] reported a study of 100 students who had completed a battery of cognitive tests selected to maximize a higher-order structure of cognitive abilities. There was the overall *g*-factor followed by primary factors of fluid (abstract), crystallized (verbal), and spatial (non-verbal) ability, all computed with *g *variance removed. Correlations between gray matter and these primary group factors were found in numerous areas distributed throughout the brain [9]. It was also the case that gray matter in some brain areas was uniquely correlated to one factor but not others, indicating the importance of separate brain networks for general and specific cognitive abilities.

In their study of 100 students, Colom et al. [15] used a hierarchical model of factor analysis to derive the *g*-factor at the highest level of factor structure and the remaining primary ability factors were computed to be independent of both *g *and of each other. This approach, based on Carroll's model [5,16], is attractive for brain research because "pure" factors may map onto separate brain networks, although there are also other factor models that may be useful [17,18]. However, independent factors constructed by psychometric techniques may not reflect the way the brain works since neural networks often have overlapping functions [19].

A complementary approach is to examine each individual test, without removing *g *variance or variance in common with other tests. This approach makes no assumptions about the psychometric structure of mental abilities and allows investigation of how an individual test score may relate to multiple brain networks. For example, two different tests of spatial ability likely share common variance due to a spatial ability factor independent of *g*, but either of the tests may show stronger and different correlations to gray matter than does the more general spatial factor. Moreover, if one of the tests measures mental rotation and the other measures spatial orientation, they may show differing patterns of brain correlates. Thus, both factor and individual test correlates of brain parameters may be informative. Note that factors reflect shared variance, whereas individual tests include additional sources of variance relevant for specific test performance.

Here we investigate how eight tests used in vocational guidance correlate to regional gray matter. Previously, we used these tests to investigate gray matter correlations to *g *and to an independent factor of spatial ability [11]. We also used these tests to investigate intelligence factors, white matter integrity, and brain function during a working memory task [20]. Here, we correlate gray matter to all the factors from that analysis and then, for the first time, we investigate gray matter correlations with each test separately. This allows a direct comparison between brain networks identified by using ability factors and networks identified by using individual tests. The main prediction is that distinguishable findings will emerge for ability factors and for individual tests because factors capture shared variance and individual tests include relevant unique variance.

## Materials and methods

### Ethics Statement

Each participant gave written informed consent as approved by the Mt. Sinai Medical Center Institutional Review Board. This research was conducted in accord with the Helsinki Declaration.

### Subjects and Procedure

During 2002-2003, 6,889 individuals sought consultation from the Johnson O'Connor Research Foundation (JOCRF), a non-profit organization dedicated to using psychometric assessments for vocational guidance. Each completed the battery of eight cognitive tests listed below in one of 11 testing centers in major U.S. cities. The mean age for all subjects was 25.4 years (*SD *= 10.6); there were 3,722 males (mean age = 25.0, *SD *= 10.2), and there were 3,207 females (mean age = 25.9, *SD *= 11.0). In addition, subjects who completed the same test battery in 2006 and 2007 in the NYC center were invited to return for MRI scanning at Mt. Sinai Medical Center. All who volunteered were screened for medical and psychiatric illnesses including a history of head injury and substance abuse. The final 40 subjects completing MRI included 21 males and 19 females, aged 18-35 years (mean age = 26.6, *SD *= 4.9).

### Cognitive Testing

The eight tests in the JOCRF battery were: Inductive Speed (IS), Analytical Reasoning (AR), Number Series (NS), Number Facility (NF), Wiggly Block (WB), Paper Folding (PF), Verbal-associative Memory (VM), and Number Memory (NM). Each is described in Additional file 1: supplemental table S1. These tests have been used in research on various aspects of cognition and intelligence [21-23]. For this study, test scores were partialled for sex and age in to eliminate nuisance variance.

### Factor Analysis

We started with all 6,929 subjects (6,889 plus the 40 with MRI scans) and followed the same procedures used by Colom et al. [15] to identify scores for groups of tests measuring distinguishable constructs. We performed a confirmatory factor analysis (CFA) on the eight test scores using the model indicated in Additional file 2: supplemental figure S1, where the resulting loadings are shown for *g *and four factors: Speed of Reasoning (Inductive Speed and Analytical Reasoning), Numerical (Number Series and Number Facility), Spatial (Wiggly Block and Paper Folding), and Memory (Verbal-associative Memory and Number Memory). Model fit was reasonable: RMSEA = .08, χ^2 ^_(16) _= 760.6, CFI = .95. This measurement model informed subsequent computations.

Next, we computed standardized scores (*z*-scores) for the eight tests shown in the measurement model (Additional file 2: supplemental figure S1) and then we computed average *z*-scores for each factor. The general intelligence *g*-score for each subject was the average of their *z*-scores on the eight tests. We then computed regression analyses using the general score (*g*) to predict Reasoning, Numerical, Spatial, and Memory, respectively. This produced residual scores for these latter factors. Additional file 3: supplemental table S2 shows the correlations between these factors and the eight tests for both the full sample of 6,929 and the 40 with MRI scans. As expected (and desired), the general score (*g*) correlated with all the tests in the battery, whereas residual scores for Speed of Reasoning, Numerical, Spatial, and Memory factors show the highest correlations with their respective measures only. Further, the *g *score is unrelated to the residual scores for the group factors. The *g *and residualized (that is, *g*-partialled) factor *z*-scores for the 40 subjects with MRI scans were used to determine the correlations to gray matter, as described below.

### Structural MRI acquisition

A 3T Siemens Allegra MRI scanner (Siemens Medical Systems, Ehrlangen, Germany) was used at Mt. Sinai Medical Center, NYC. For each subject, a sagittal T_1_-weighted spin echo image was performed first as localizer, with the repetition time (TR) = 500 msec and the echo time (TE) = 10 msec, FOV = 18cm × 14 cm, matrix size = 512 × 384, 4.3 mm thick. Based on this localizer, structural scans were acquired using a 3 D MP-RAGE pulse sequence with the following parameters: TR = 2500 ms, TE = 4.4 ms, FOV = 21 cm, matrix size = 256 × 256, 208 slices with thickness = 0.82 mm.

### Voxel-based-morphometry (VBM) and statistical analyses

We applied VBM to identify brain areas where gray matter (GM) volumes are correlated to the scores of interest. We used Statistical Parametric Mapping software (SPM5; The Wellcome Department of Imaging Neuroscience, University College London) to apply the VBM unified segmentation protocol [24-26]. This included bias correction and, to preserve the amount of tissue in any given anatomical region after spatial normalization, the optimal GM partitions were multiplied by the Jacobian determinants of their respective spatial transformation matrix. This step allows the final VBM statistics to reflect local deviations in the absolute amount (volume) of tissue in different regions of the brain [24]. The modulated GM partitions were smoothed (12-mm FWHM isotropic Gaussian kernel) to account for slight misalignments of homologous anatomical structures and to ensure statistical validity under parametric assumptions. Each individual scan was fitted to a standardized SPM template created for 3T MRI scans (tissue probability map provided by the International Consortium for Brain Mapping (T1 452 Atlas, J. C. Mazziotta & A. W. Toga, http://www.loni.ucla.edu/Atlases/Atlas_Detail.jsp?atlas_id=6). The General Linear Model was used in the SPM analyses and age and sex were entered as nuisance variables. Given the limited statistical power of 40 subjects, we detail results at p < .001, uncorrected, in all the tables and provide best estimates of Brodmann areas (BA) for anatomical localization using the Talairach & Tourneau Brain Atlas co-ordinates [27]; figures are shown consistently for all analyses at p < .01 uncorrected, to allow straightforward comparisons. Findings corrected using the False Discovery Rate (FDR) p < .05 are noted; no findings survived correction using Family Wise Error (FWE).

## Results

### Gray matter correlations with factor scores

**Figure 1:**
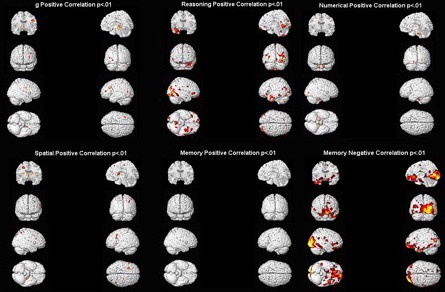
**Gray matter correlations with factor scores**. Correlations between gray matter and scores on each intelligence factor (see Table 1).

As shown in figure 1 and detailed in table 1, the Speed of Reasoning factor shows the most positive correlations with gray matter in areas distributed throughout the brain including posterior cingulate BA 31, BA 37/38 in the temporal lobe and frontal BAs 10 and 47. There is no overlap with any of the other factors, all of which show few if any areas correlated to gray matter (p < .001, table 1; see Additional file 4: supplemental figure S2 for a representative scatterplot). Also shown in figure 1 (lower right), only the Memory factor shows any systematic negative correlations (p < .05 FDR corrected; see table 1), where less gray matter is associated with higher scores. These areas (table 1) include a large cluster in the occipital lobe (BAs 17, 18, 19; p < .025 FDR corrected), the cingulate gyrus (BAs 31, 32), the post central gyrus (BAs 3, 43) and frontal BAs 10, 11, 46, 47.

**Table 1:** Brain areas with significant gray matter correlations (p < .001 uncorrected) with each intelligence factor (all correlations are positive except for the Memory factor)*

Factor Name	B A	Region Name	x	y	z	Z	Cluster	FDR
*g*-factor (+)	BA 9	Middle Frontal Gyrus	-36	23	30	4.02	165	

		Substania Nigra	12	-22	-7	3.17	272	

								

Speed of Reasoning (+)	BA 37	Sub-Gyral	48	-45	-5	3.47	895	

	BA 47	Inf. Frontal Gyrus	-50	42	-16	3.08	35	

	BA 10	Inf. Frontal Gyrus	57	41	-2	3.07	66	

	BA 38	Sup. Temporal Gyrus	-42	5	-17	3.03	416	

	BA 31	Posterior Cingulate	-24	-63	14	3.03	700	

	BA 18	Lingual Gyrus	30	-74	-8	2.97	147	

		Inf. Semi-Lunar Lob.	6	-65	-49	3.59	598	

								

Numerical Factor (+)	BA 18	Mid. Occipital Gyrus	26	-81	6	3.16	76	

	BA 24	Cingulate Gyrus	-12	2	31	3.03	33	

								

Spatial Factor (+)	BA 8	Mid. Frontal Gyrus	-30	14	40	3.64	186	

		Lat. Geniculum Body	-26	-23	-2	3.03	178	

								

Memory Factor (-)	BA 17	Cuneus	24	-91	1	4.29	12,856	.025

	BA 19	Mid. Occipital Gyrus	28	-81	15	4.21		.025

	BA 18	Lingual Gyrus	14	-68	5	4.21		.025

	BA 31	Cingulate Gyrus	20	-47	23	3.66	136	.025

	BA 37	Sub-Gyral	-50	-45	-8	3.52	371	.025

	BA 47	Inf. Frontal Gyrus	-50	38	-17	3.51	196	.028

	BA 32	Anterior Cingulate	10	30	-12	3.46	5,131	.030

	BA 11	Sup. Frontal Gyrus	-16	54	-15	3.30		.037

	BA 11	Med. Frontal Gyrus	-6	61	-17	3.28		.037

	BA 21	Mid. Temporal Gyrus	-44	2	-34	3.27	737	.038

	BA 38	Sup. Temporal Gyrus	-34	6	-27	3.03		

	BA 3	Postcentral Gyrus	57	-18	29	2.74		

	BA 10	Mid. Frontal Gyrus	-30	52	1	3.13	140	.046

	BA 46	Inf. Frontal Gyrus	55	43	2	3.11	104	.048

	BA 43	Postcentral Gyrus	69	-9	17	2.98	136	

		Thalamus	-24	-23	1	3.01	752	

		Thalamus	-16	-31	2	2.98		

* BA is Brodmann Area, Talairach x, y, z co-ordinates; positive × values are in right hemisphere; Z is z-score; cluster size is number of voxels (blank entry denotes part of previous cluster); FDR is False Discovery Rate (blank entry denotes not significant p < .05).

### Gray matter correlations with individual tests

**Figure 2:**
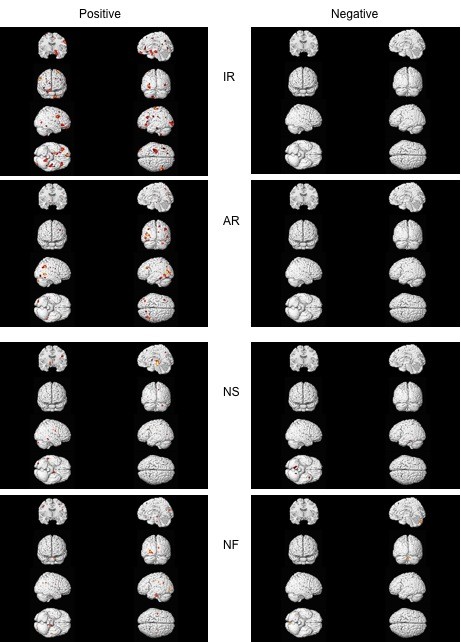
**Gray matter correlations with test scores comprising Speed of Reasoning and Numerical factors**. Correlations between gray matter and scores on Inductive Reasoning (IR) and Analytical Reasoning (AR) tests comprising the Speed of Reasoning Factor, and scores on Number Series (NS) and Number Facility (NF) tests comprising the Numerical Factor; see Additional files 5 and 6: Supplemental Tables S3 and S4.

**Figure 3:**
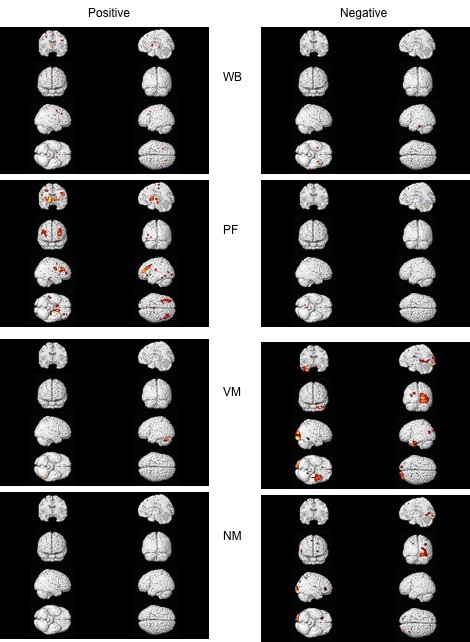
**Gray matter correlations with test scores comprising Spatial and Memory factors**. Correlations between gray matter and scores on Wiggly Block (WB) and Paper Folding (PF) tests comprising the Spatial Factor, and scores on Verbal-associative Memory (VM) and Number Memory (NM) comprising the Memory Factor; see Additional files 7 and 8: supplemental tables S5 and S6.

Correlations for each of the eight tests (two per factor) are shown in figures 2 and 3 and detailed in additional files 5, 6, 7, 8 (p < .001 uncorrected; no findings survived FDR or FWE correction). For the Speed of Reasoning factor, the IR test (figure 2 top and Additional file 5: supplemental table S3) shows positive gray matter correlations mostly in frontal areas, but for the AR test, there are half as many areas, mostly posterior. There are no negative correlations. These patterns suggest that the different reasoning tests are related to different brain networks although the Speed of Reasoning factor correlations show a combination of both patterns.

The two tests comprising the Numerical factor (NS and NF) both show different, but weak patterns positively correlated to gray matter (figure 2 bottom and Additional file 6: supplemental table S4). NS had a cluster in the thalamus; NF had clusters in occipital BA 18.

The spatial factor was based on the WB and PF tests. Whereas the WB test showed only one small area of positive and one small area of negative correlations with gray matter, the PF test showed many areas with positive correlations (figure 3 top and Additional file 7: supplemental table S5). These were distributed cortically and subcortically. They include a large cluster in the frontal lobe (BAs 10, 9, 6), a cluster around the globus pallidus, the caudate, a large midbrain cluster, and a large cluster in the cerebellum. The PF findings alone are stronger than the Spatial factor findings.

Both tests comprising the Memory factor show numerous negative correlations with gray matter. VM scores show a large cluster in the occipital lobe (BA 18, 19), the middle temporal lobe (BA 38) and the posterior cingulate (BA 30) (figure 3 top; Additional file 8: supplemental table S6). The NM test scores show a different pattern with clusters both in occipital (BAs 17, 18, 19) and frontal lobes (BAs 11, 45, 8) (figure 3 bottom, Additional file 8: supplemental table S6). These patterns have some overlap with each other and both contribute to the strong pattern of gray matter negative correlations seen with the Memory factor.

## Discussion

Scores from individual tests offer some advantages in the practice of vocational guidance. In addition to being more transparent and related to specific performance than factor scores, individual tests also can provide measurement of more-specific abilities than broader factors allow. The results of this study suggest that patterns of brain correlates may be distinct for tests of specific abilities and factors. These observations are based on qualitative comparisons but they illustrate the potential value of examining separate indices of performance. There are also meaningful results at the levels of group factors and *g*, and so it appears that analysis of all three levels is important for understanding brain/cognitive relationships [28]. The results for *g *are consistent with other findings, especially the P-FIT model of brain areas hypothesized to underlie general intelligence [9] and are detailed elsewhere [11].

Specifically for the other factors, in this sample, Speed of Reasoning and Memory showed relatively strong gray matter correlates. The two individual tests for the Speed of Reasoning factor showed different patterns and both contributed to the factor pattern. For the Memory factor, both tests showed similar results. One test in the Spatial factor was informative (PF) and the other not so much. Neither test in the Numerical factor showed informative gray matter correlates.

The inverse direction of the gray matter correlations for the Memory factor was evident in both component tests, although we are unaware of any previous reports of inverse correlations between gray matter and other similar tests. No individual subjects showed anomalies that could account for this direction of relationship. Inverse correlations between performance on some cognitive tests and functional imaging has been interpreted as evidence for the importance of efficient use of neural resources, and it has been hypothesized that efficient function may result from more gray matter [9]. There is no specific evidence, however, that this is the case here [20]. Since there are previous reports of sex differences in the patterns of gray matter correlates to intelligence test scores [29-31], we recomputed these analyses for males and females separately. Only the males showed the inverse pattern. Why this should be the case is not clear. Lynn and Irwing [32] reported a small average advantage for males in two memory measures after the analysis of large samples taken from worldwide standardizations of the Wechsler scales (WPPSI, WISC, and WAIS). This small difference could reflect different brain correlates for the memory factor but it does not explain the inverse correlations. Since the sample sizes, however, were quite small for VBM stability (21 males, 19 females), we cannot interpret this finding with confidence. In general, VBM requires larger samples than 40 for stability, so this report is offered as an exploratory account of factor versus test correlates with gray matter in a sample uniquely characterized with a comprehensive test battery.

In summary, individual test results suggested some degree of consistency with their respective factors, but also some differences. Separating sources of variance contributing to participants' performance on intelligence measures is especially important [11]. The influence of *g *is pervasive, but it changes for different group (lower order) factors and individual tests. Participants' scores result from *g*, broad cognitive abilities (group factors), and specific cognitive skills (test specificities). Brain correlates for any given cognitive performance are influenced by all these sources of variance. We did not have sufficient statistical power in this pilot study to determine these different contributions, but our results suggest that, while individual tests share some relevant variance with their corresponding ability factors, they also may have informative uniqueness for understanding underlying brain networks.

## Competing interests

This project was funded at the Mt. Sinai Medical Center (Cheuk Tang), New York City, by the non-profit Johnson O'Connor Research Foundation (JOCRF). Cheuk Tang and Kevin Head received partial salary support. David Schroeder is an employee of JOCRF and Richard Haier was a paid consultant. The JOCRF did not have any institutional approval or supervisory role in the preparation of the manuscript or the decision to publish. Roberto Colom is funded by grants SEJ-2006-07890 and PR2008-0038 (Ministerio de Ciencia e Innovacion, MICINN).

## Authors' contributions

RJH and DHS conceived and designed the study. CT collected the imaging data. KH, DHS and RC analyzed the data. All authors approved and helped write the manuscript.

## Supplementary Material

Additional file 1 **Description of the eight cognitive tests**. Supplemental table S1. Click here for file

Additional file 2 **Factor Structure of the test battery according to a confirmatory factor analysis (N = 6929)**. Supplemental figure S1. Click here for file

Additional file 3 **Correlations between factors and the eight tests for both the full sample of 6,929 and the 40 with MRI scans**. Supplemental table S2. Click here for file

Additional file 4 **Scatterplot showing the general factor (*g*) correlation with gray matter in BA9 (normalized scores; N = 40; see table **1** for maximum voxel location)**. Supplemental figure S2. Click here for file

Additional file 5 **Gray matter correlations with IR and AR**. Supplemental table S3. Click here for file

Additional file 6 **Gray matter correlations with NS and NF**. Supplemental table S4. Click here for file

Additional file 7 **Gray matter correlations with WB and PF**. Supplemental table S5. Click here for file

Additional file 8 **Gray matter correlations with VA and NM**. Supplemental table S6. Click here for file
