# Supplementary material for: Gray matter correlates of cognitive ability tests used for vocational guidance
Source: BMC Res Notes. 2010 Jul 22;3:206. doi: 10.1186/1756-0500-3-206 (PMC2917438; doi:10.1186/1756-0500-3-206)
Supplement: Additional file 1 — Description of the eight cognitive tests. Supplemental table S1. [file 1756-0500-3-206-S1.DOC]

Supplemental Table 1. Johnson O'Connor Research Foundation (JOCRF) Test Battery

| **Test name** | **Reliability** | **Ability measured** | **Description of task** |
| --- | --- | --- | --- |
|  |  |  |  |
| Inductive Speed (IS) | 0.84 | Quickness in seeing relationships among separate facts, ideas, or observations. | Given six pictures, quickly identifying the three pictures that go together (highly speeded). |
|  |  |  |  |
| Analytical Reasoning (AR) | 0.81 | Ability to arrange ideas into a logical sequence. | Given a set of words, placing them into a predetermined logical structure so that they make sense (e.g., ANIMAL: DOG, CAT). |
|  |  |  |  |
| Number Series (NS) | 0.87 | Ability to reason (solve problems) with numbers. | Given a series of numbers, identifying the number that would come next in the sequence. |
|  |  |  |  |
| Number Facility (NF) | 0.86 | Ability to perform arithmetic operations quickly. | Given six numbers, placing them into two simple equations so that the equations are true. |
|  |  |  |  |
| Wiggly Block (WB) | 0.73 | Ability to visualize three-dimensional forms. | Re-assembling three-dimensional blocks that have been cut into wavy (“wiggly”) pieces. |
|  |  |  |  |
| Paper Folding (PF) | 0.82 | Ability to visualize three-dimensional forms. | Mentally visualizing a piece of paper as it is folded, punched with a paper punch, and unfolded. |
|  |  |  |  |
| Verbal-Associative Memory (VM) | 0.92 | Associative memory for verbal material. | Memorizing paired associates between nonsense words and English words. |
|  |  |  |  |
| Number Memory (NM) | 0.82 | Memory for numbers. | Memorizing six-digit numbers. |
