# Supplementary figures and images for: Gray matter correlates of cognitive ability tests used for vocational guidance
Source: BMC Res Notes. 2010 Jul 22;3:206. doi: 10.1186/1756-0500-3-206 (PMC2917438; doi:10.1186/1756-0500-3-206)

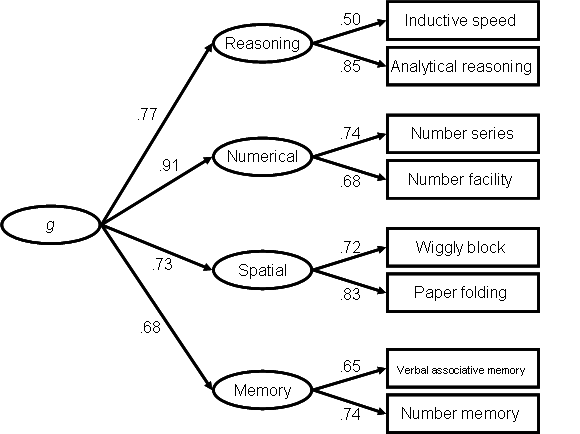

Supplement: Additional file 2 — Factor Structure of the test battery according to a confirmatory factor analysis (N = 6929). Supplemental figure S1. [file 1756-0500-3-206-S2.TIFF]

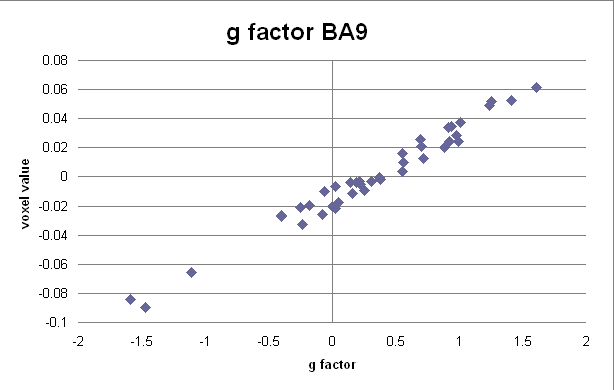

Supplement: Additional file 4 — Scatterplot showing the general factor (g) correlation with gray matter in BA9 (normalized scores; N = 40; see table 1 for maximum voxel location). Supplemental figure S2. [file 1756-0500-3-206-S4.TIFF]
