# Supplementary material for: Gray matter correlates of cognitive ability tests used for vocational guidance
Source: BMC Res Notes. 2010 Jul 22;3:206. doi: 10.1186/1756-0500-3-206 (PMC2917438; doi:10.1186/1756-0500-3-206)
Supplement: Additional file 3 — Correlations between factors and the eight tests for both the full sample of 6,929 and the 40 with MRI scans. Supplemental table S2. [file 1756-0500-3-206-S3.DOC]

Supplemental Table 2

Correlations (full sample/MRI sample) between factors (with *g* removed) and tests

|  | **General** | **Reasoning** | **Numerical** | **Spatial** | | **Memory** | |
| --- | --- | --- | --- | --- | --- | --- | --- |
| Tests |  |  |  | |  | |  |
| Inductive speed | .53**/.38* | .66**/.75** | -.22**/-.23 | | -.16**/-.29 | | -.27**/-.31* |
| Analytical reason. | .71**/.81** | .48**/.23 | -.13**/.13 | | -.09**/-.14 | | -.25**/-.25 |
| Number series | .70**/.83** | -.24**/-.33* | .51**/.56** | | -.14**/-.16 | | -.08**/-.06 |
| Number facility | .67**/.72** | -.08**/-.20 | .55**/.72** | | -.27**/-.49** | | -.14**/-.10 |
| Wiggly block | .65**/.71** | -.08**/-.20 | -.25**/-.05 | | .61**/.39* | | -.29**/-.09 |
| Paper folding | .69**/.69** | -.18**/.24 | -.20**/-.10 | | .57**/.46** | | -.20**/-.07 |
| Verbal-assoc mem. | .58**/.71** | -.25**/-.36* | -.15**/-.02 | | -.29**/-.12 | | .64**/.52** |
| Number memory | .62**/.58** | -.31**/-.39* | -.10**/.05 | | -.23**/-.29 | | .60**/.62** |

** p* < .05, ** *p* < .01
