# Supplementary material for: Gray matter correlates of cognitive ability tests used for vocational guidance
Source: BMC Res Notes. 2010 Jul 22;3:206. doi: 10.1186/1756-0500-3-206 (PMC2917438; doi:10.1186/1756-0500-3-206)
Supplement: Additional file 5 — Gray matter correlations with IR and AR. Supplemental table S3. [file 1756-0500-3-206-S5.DOC]

Supplemental Table 3. Brain areas with significant gray matter correlations

with IR and AR tests comprising the Reasoning factor (all positive, p<.001, uncorrected, N=40)*

| **Test** | **Z** | **Cluster** | **x** | **y** | **z** | **Location** | **BA** |
| --- | --- | --- | --- | --- | --- | --- | --- |
| Inductive Reasoning | 3.72 | 217 | 63 | 11 | 31 | Inferior Frontal Gyrus | BA 9 |
|  | 3.69 | 198 | -28 | -12 | 71 | Precentral Gyrus | BA 6 |
|  | 3.29 | 202 | -36 | 29 | -8 | Inferior Frontal Gyrus | BA 47 |
|  | 3.2 | 175 | -38 | 23 | 26 | Middle Frontal Gyrus | BA 9 |
|  | 3.07 | 324 | -34 | -97 | 0 | Middle Occipital Gyrus | BA 18 |
|  | 3.03 | 145 | -14 | 23 | 32 | Cingulate Gyrus | BA 32 |
|  | 2.99 | 125 | 14 | 53 | -25 | Superior Frontal Gyrus | BA 11 |
|  | 2.97 | 313 | 40 | 33 | -5 | Inferior Frontal Gyrus | BA 47 |
|  | 2.99 | 187 | 14 | -20 | -11 | Substantia Nigra |  |
|  |  |  |  |  |  |  |  |
| Analytical Reasoning | 3.81 | 128 | -36 | 25 | 28 | Middle Frontal Gyrus | BA 9 |
|  | 3.57 | 262 | -42 | -71 | 16 | Middle Temporal Gyrus | BA 39 |
|  | 3.14 | 166 | 50 | -52 | 4 | Middle Temporal Gyrus | BA 37 |
|  | 3.05 | 125 | -46 | -39 | -11 | Fusiform Gyrus | BA 37 |

*Z is z-score, Cluster is size (number of voxels; blank entry denotes part of previous cluster), x, y, z co-ordinates in Talairach space, BA is Brodmann Area
