# Supplementary material for: Gray matter correlates of cognitive ability tests used for vocational guidance
Source: BMC Res Notes. 2010 Jul 22;3:206. doi: 10.1186/1756-0500-3-206 (PMC2917438; doi:10.1186/1756-0500-3-206)
Supplement: Additional file 6 — Gray matter correlations with NS and NF. Supplemental table S4. [file 1756-0500-3-206-S6.DOC]

Supplemental Table 4. Brain areas with significant gray matter correlations

with the NS and NF tests comprising the Numerical factor (p<.001, uncorrected, N=40)*

| **Test** | **Z** | **Cluster** | **x** | **y** | **z** | **Location** | **BA** |
| --- | --- | --- | --- | --- | --- | --- | --- |
| Number Series (+) | 3.10 | 711 | 4 | -25 | 1 | Thalamus |  |
|  |  |  |  |  |  |  |  |
| Number Series (-) | 3.99 | 328 | 16 | -59 | -53 | Cerebellum |  |
|  | 2.98 |  | -16 | -57 | -53 | Cerebellum |  |
|  |  |  |  |  |  |  |  |
| Number Facility (+) | 3.16 | 182 | -26 | -83 | 6 | Mid. Occipital Gyrus | BA 18 |
|  | 3.00 | 49 | -12 | -27 | -27 | Culmen |  |
|  | 2.97 | 35 | 14 | -80 | 24 | Cuneus | BA 18 |

*Z is z-score, Cluster is size (number of voxels; blank entry denotes part of previous cluster), x, y, z co-ordinates in Talairach space, BA is Brodmann Area
