# Supplementary material for: Gray matter correlates of cognitive ability tests used for vocational guidance
Source: BMC Res Notes. 2010 Jul 22;3:206. doi: 10.1186/1756-0500-3-206 (PMC2917438; doi:10.1186/1756-0500-3-206)
Supplement: Additional file 7 — Gray matter correlations with WB and PF. Supplemental table S5. [file 1756-0500-3-206-S7.DOC]

Supplemental Table 5. Brain areas with significant gray matter correlations

with the WB and PF tests comprising the Spatial factor (p<.001, uncorrected, N=40)*

| **Test** | **Z** | **Cluster** | **x** | **y** | **z** | **Location** | **BA** |
| --- | --- | --- | --- | --- | --- | --- | --- |
| Wiggly Block (+) | 3.58 | 118 | 28 | 27 | 32 | Mid. Frontal Gyrus | BA 9 |
|  |  |  |  |  |  |  |  |
| Wiggly Block (-) | 3.81 | 150 | -38 | 10 | -41 | Mid. Temporal Gyrus | BA 38 |
|  |  |  |  |  |  |  |  |
| Paper Folding (+) | 4.39 | 1223 | -30 | 43 | 11 | Mid. Frontal Gyrus | BA 10 |
|  | 4.25 |  | -36 | 27 | 30 | Mid. Frontal Gyrus | BA 9 |
|  | 3.13 |  | -30 | 10 | 40 | Mid. Frontal Gyrus | BA 6 |
|  | 3.83 | 1285 | -12 | 4 | 5 | Lat. Globus Pallidus |  |
|  | 3.56 | 1505 | -14 | -30 | -10 | Culmen |  |
|  | 3.24 |  | 16 | -22 | -4 | Substania Nigra |  |
|  | 3.12 |  | -14 | -25 | 0 | Thalamus |  |
|  | 3.50 | 596 | 36 | 36 | 22 | Mid. Frontal Gyrus | BA 10 |
|  | 3.27 |  | 40 | 28 | 24 | Mid. Frontal Gyrus | BA 46 |
|  | 3.46 | 348 | 44 | -4 | 28 | Precentral Gyrus | BA 6 |
|  | 3.37 | 520 | 12 | 4 | 7 | Caudate Body |  |
|  | 3.00 | 91 | 32 | 47 | 5 | Mid. Frontal Gyrus | BA 10 |
|  | 3.00 | 51 | -48 | -56 | -2 | Inf. Temporal Gyrus | BA 19 |
|  |  |  |  |  |  |  |  |
| Paper Folding (-) | 3.53 | 74 | 12 | -63 | -52 | Cerebellum |  |

*Z is z-score, Cluster is size (number of voxels; blank entry denotes part of previous cluster), x, y, z co-ordinates in Talairach space, BA is Brodmann Area
