# Supplementary material for: Gray matter correlates of cognitive ability tests used for vocational guidance
Source: BMC Res Notes. 2010 Jul 22;3:206. doi: 10.1186/1756-0500-3-206 (PMC2917438; doi:10.1186/1756-0500-3-206)
Supplement: Additional file 8 — Gray matter correlations with VA and NM. Supplemental table S6. [file 1756-0500-3-206-S8.DOC]

Supplemental Table 6. Brain areas with significant gray matter correlations

with the VA and NM tests comprising the Memory factor (p<.001, uncorrected, N=40)*

| **Test** | **Z** | **Cluster** | **x** | **y** | **z** | **Location** | **BA** |
| --- | --- | --- | --- | --- | --- | --- | --- |
| Verbal-asso. Mem (+) | 3.03 | 230 | -55 | -63 | -20 | Cerebellum |  |
|  |  |  |  |  |  |  |  |
| Verbal-asso. Mem (-) | 3.88 | 953 | -28 | -69 | 16 | Posterior Cingulate | BA 30 |
|  | 3.34 |  | -20 | -48 | 6 | Parahippocampus | BA 30 |
|  | 3.66 | 1162 | -36 | 10 | -36 | Mid. Temporal Gyrus | BA 38 |
|  | 3.31 |  | -40 | -5 | -25 | Mid. Temporal Gyrus | BA21 |
|  | 3.47 | 2871 | 44 | -89 | -2 | Inf. Occipital Gyrus | BA 18 |
|  | 3.34 |  | 26 | -83 | 17 | Mid. Occipital Gyrus | BA 19 |
|  | 3.29 |  | 32 | -89 | -2 | Mid. Occipital Gyrus | BA 18 |
|  | 3.21 | 148 | -16 | -94 | 30 | Cuneus | BA 19 |
|  | 3.07 | 56 | -16 | -47 | -51 | Cerebellum |  |
|  | 3.05 | 22 | 18 | -17 | 58 | Precentral Gyrus | BA 6 |
|  |  |  |  |  |  |  |  |
| Number Memory (-) | 3.67 | 996 | 40 | -93 | 6 | Mid. Occipital Gyrus | BA 19 |
|  | 3.31 |  | 20 | -90 | -6 | Inf. Occipital Gyrus | BA 17 |
|  | 3.31 |  | 42 | -91 | -4 | Inf. Occipital Gyrus | BA 18 |
|  | 3.29 | 422 | 16 | -66 | 5 | Lingual Gyrus | BA 19 |
|  | 3.09 | 71 | 57 | 41 | 2 | Inf. Frontal Gyrus | BA 45 |
|  | 2.98 | 29 | 10 | 47 | 49 | Sup. Frontal Gyrus | BA 8 |
|  | 2.97 | 103 | 24 | 24 | -26 | Inf. Frontal Gyrus | BA 11 |

*Z is z-score, Cluster is size (number of voxels; blank entry denotes part of previous cluster), x, y, z co-ordinates in Talairach space, BA is Brodmann Area
